# Supplementary material for: Break-induced replication is enhanced by a phospho-activated RPA-binding module in Pol32
Source: Nat Commun. 2026 Apr 9;17:5006. doi: 10.1038/s41467-026-71309-y (PMC13237216; doi:10.1038/s41467-026-71309-y)
Supplement: Supplementary file 1 — Supplementary Information [file 41467_2026_71309_MOESM1_ESM.pdf]

Supplementary Information

Break-induced replication is enhanced by a phospho-activated RPA-binding module in Pol32

David Jones*, Rowin Appanah*, Luke A Yates, Antony W Oliver✉, Ulrich Rass✉

✉email: Antony.Oliver@sussex.ac.uk; U.W.Rass@sussex.ac.uk

This file includes:
Supplementary Figures 1-9
Supplementary Tables 1, 2

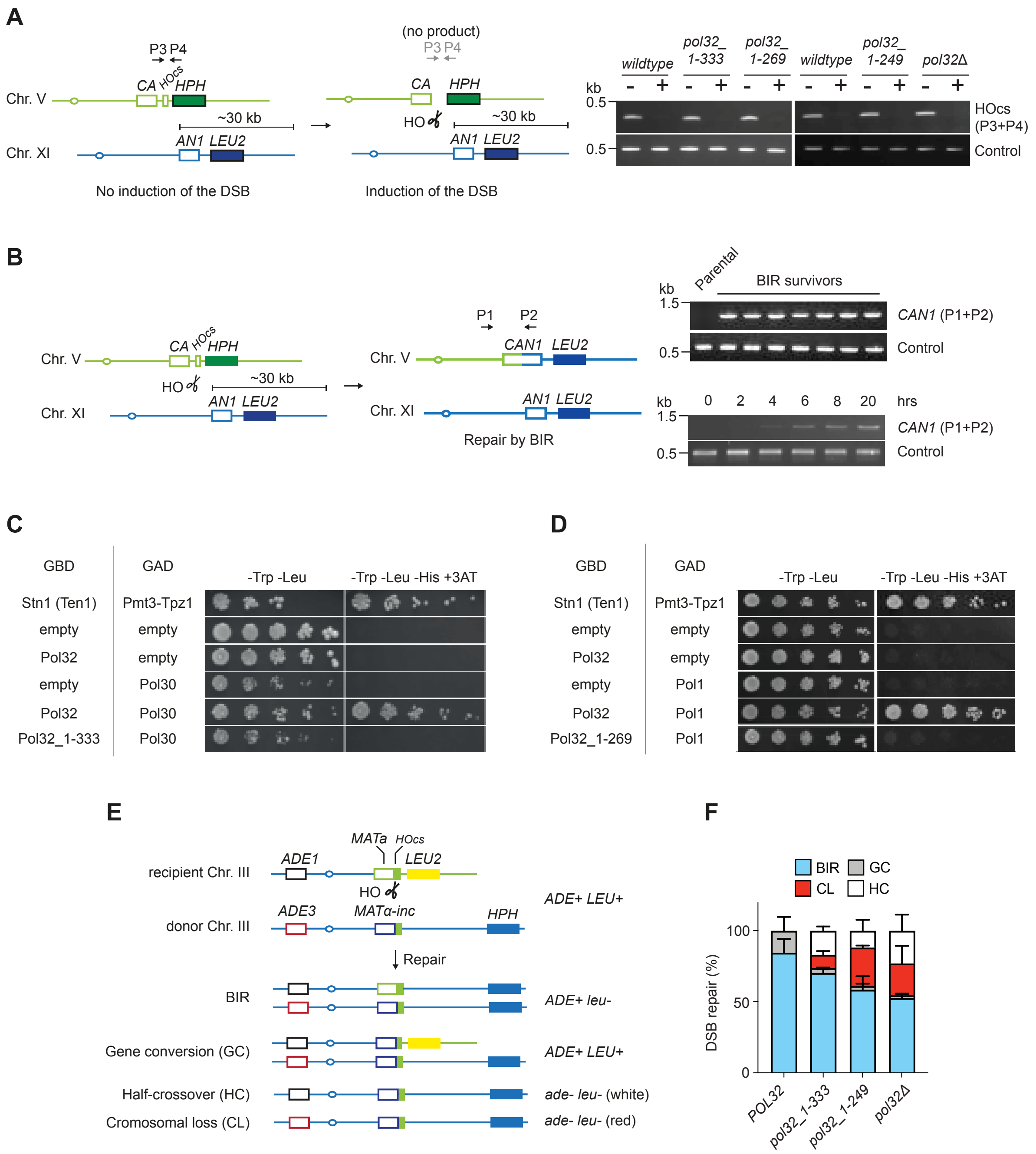

Supplementary Figure 1 | Analysis of Pol32 by BIR and yeast two-hybrid assays. (A) Cleavage of *HOcs* determined for the indicated strains upon galactose-mediated induction (+) or not (-) of the HO endonuclease for 2 h (n = 1). PCR across *HOcs* with primers P3 and P4 on equal amounts of genomic DNA. The positions of size markers (kb) are indicated (B) Restoration of *CAN1* by BIR after *HOcs* cleavage in JRL-derived strains. Top right, seven surviving colonies from JRL092 cells plated on YPGAL medium were randomly selected for PCR (primers P1 and P2) on genomic DNA to monitor restoration of *CAN1* (n = 1). Bottom right, *CAN1* restoration by BIR over 20 h after galactose-mediated HO endonuclease induction (representative example of n = 3 biological repeats). The positions of size markers (kb) are indicated. Control; PCR on an unrelated locus (*CDC5*). (C, D) Yeast two-hybrid analysis of the indicated plasmid-borne Pol32 constructs fused to the Gal4-binding domain (GBD) for interaction with Pol30 (PCNA) and Pol1 (Pol α) fused to the Gal4-activating domain (GAD). Strains harbouring the listed constructs were spotted in serial dilutions (n = 1). Growth on plates lacking tryptophan and leucine (-Trp -Leu) indicates the presence of both a GBD and GAD plasmid, while growth on plates lacking tryptophan, leucine and histidine, and containing

3-amino-1,2,4-triazole (-Trp -Leu -His +3AT) indicates a protein interaction. Fission yeast Stn1 (Ten1) and Pmt3-Tpz1 served as positive control. (E) Schematic representation of the allelic BIR assay. Premature or aberrant processing of BIR intermediates results in half-crossovers (HC) or chromosomal loss (CL). (F) Allelic BIR assay for the indicated strains grown to logarithmic phase in YP-raffinose and plated onto YPGAL. Surviving colonies were replica-plated on appropriate selective media to classify repair outcomes (mean of n = 3 independent biological repeats, SD indicated). Source data are provided as a Source Data file.

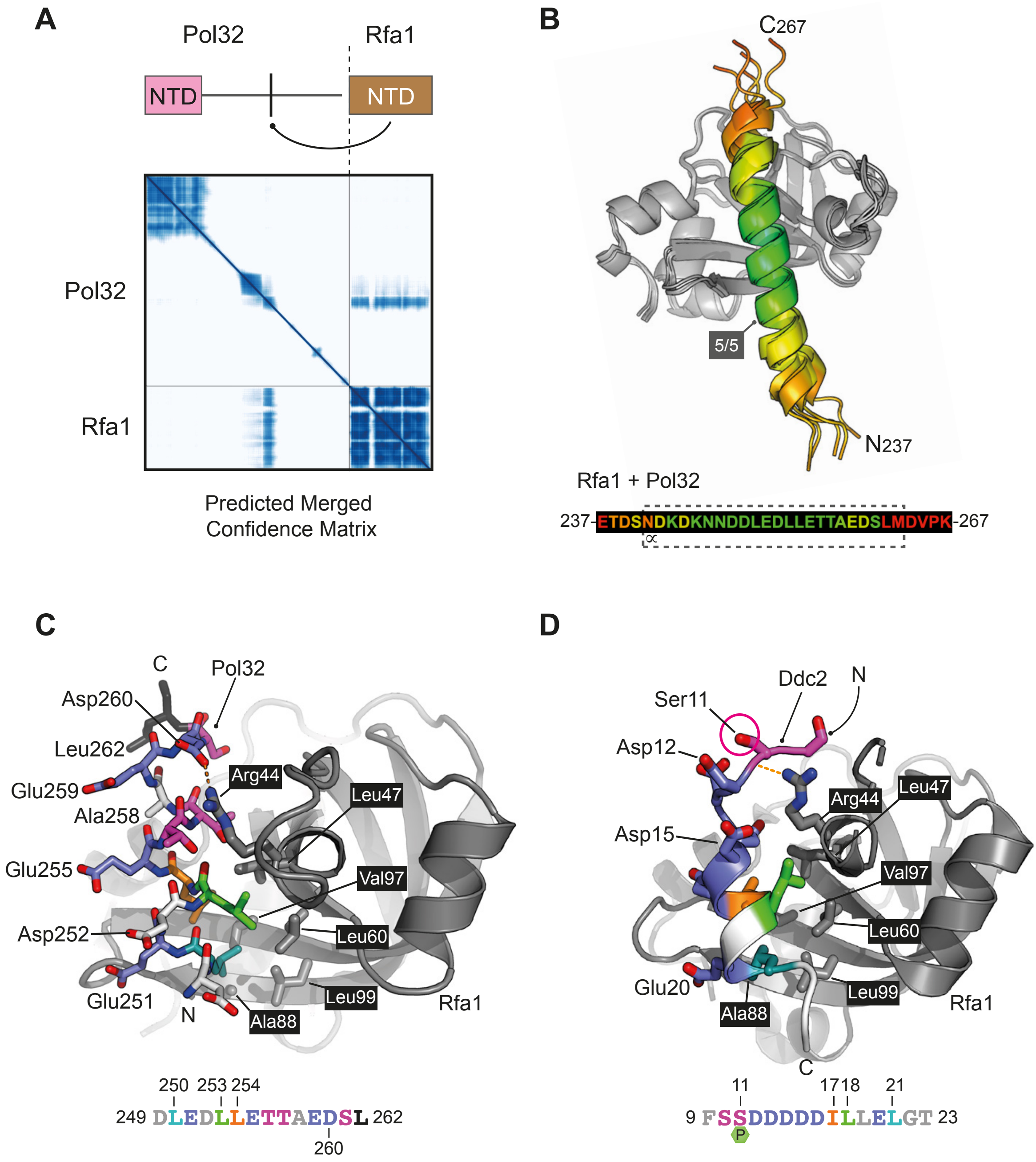

Supplementary Figure 2 | Molecular models for Pol32-RPA interactions. (A) Predicted Merged Confidence Matrix, as generated by AlphaBridge, for the interaction between full-length Pol32 and Rfa1 NTD. The arrow indicates binding of the Rfa1 NTD to the region of Pol32 containing the suspected RBM. (B) Superposition of five independent AlphaFold models for Pol32 in complex with the Rfa1 NTD. Models are shown in cartoon representation with Rfa1 NTD rendered grey and the identified interacting region of Pol32 spanning amino acid residues 237 to 267 (see inset) coloured according to pLDDT (predicted Local Distance Difference Test) score, using a continuous rainbow spectrum from red (low confidence) to blue (high confidence). An interacting α-helical segment of Pol32 comprising amino acids Asn241-Met263 was predicted with high consistency (see inset: indicated with a dotted outline and labelled 'α'). (C) Molecular cartoon representation of the predicted interface between Rfa1 NTD and Pol32. Key amino acids are shown in stick representation and are colour-coded as per Figure 2A. (D) Molecular cartoon representation of the X-ray crystal structure of Rfa1 NTD in complex with a peptide derived from Ddc2. Key amino acids are shown in stick representation and are colour-coded as per Figure 2A. For simplicity and to aid comparison, the phosphorylation at Ser11 is not shown.

A

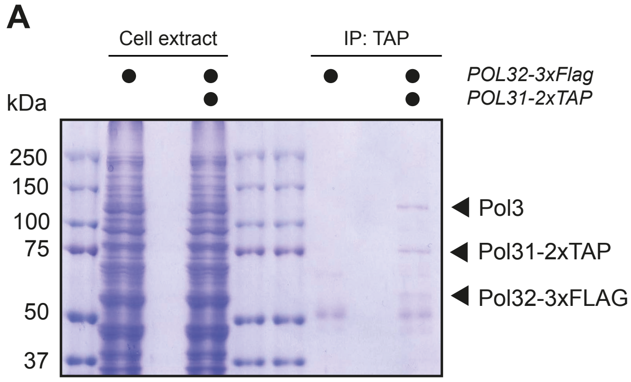

B

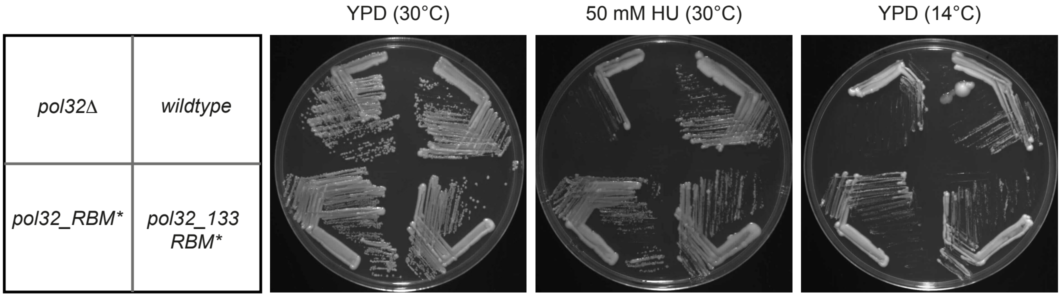

Supplementary Figure 3 | Affinity capture of the Pol δ complex and replication competence of Pol32 RBM-mutant cells. (A) Representative co-immunoprecipitation of the Pol δ holocomplex by TAP-IP targeting Pol31-TAP (n = 2 biological repeats). A strain harbouring untagged Pol31 served as control. Input (cell extract, 1/10 loaded) and IP samples were resolved by SDS-polyacrylamide gel electrophoresis and proteins stained with Coomassie blue. Protein bands specifically enriched with Pol31-TAP represent Pol32-Flag and Pol3 as indicated. The positions of size markers (kDa) are shown on the left. (B) Representative steak-outs of cells harbouring Pol32 RBM* and PIP-box/RBM* mutants (n = 2 biological repeats), which, in contrast to *pol32*Δ cells, exhibit no overt replication stress or cold sensitivity indicating that canonical Pol δ DNA replication functions are preserved.

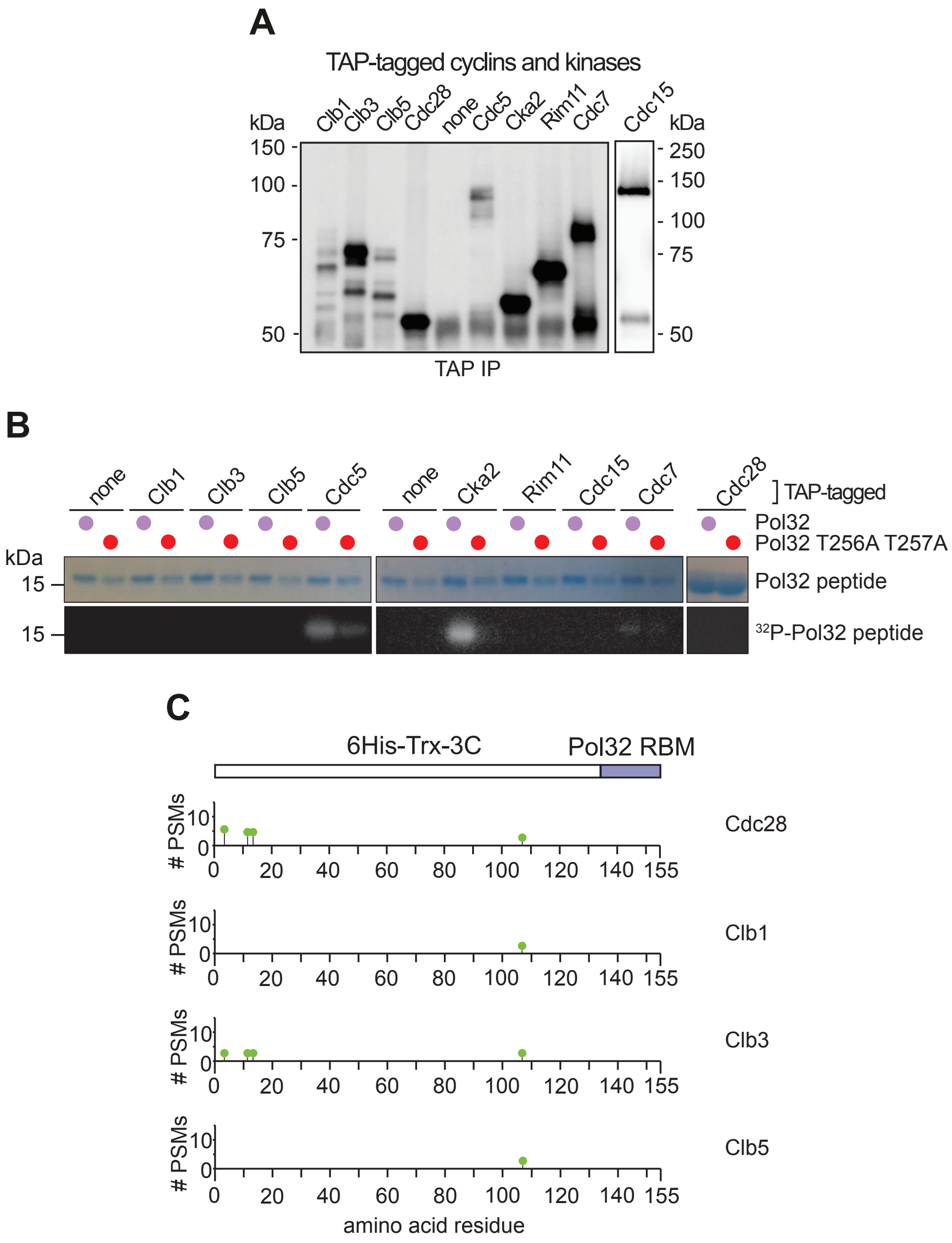

Supplementary Figure 4 | Kinase assays with affinity-captured protein kinases on Pol32 RBM peptides. (A) Representative immunoprecipitation of the indicated TAP-tagged protein kinases analysed by western blotting (anti-TAP; n = 2 biological repeats). The positions of size markers (kDa) are indicated. Cells were harvested in logarithmic phase except those expressing Cdc28-TAP, Clb1-TAP and Clb3-TAP, which were harvested after synchronisation in G2/M, and those expressing Clb5-TAP, which were harvested in S-phase 20 min after release from G1 arrest. (B) Affinity captured kinases where incubated with Trx-Pol32 RBM peptides as indicated in the presence of ATP [γ-^{32}P] and analysed by agarose gel electrophoresis and phosphor-imaging to monitor modification by ^{32}P (n = 1). The positions of size markers (kDa) are indicated. (C) The annotated affinity captured kinases where incubated with Trx-Pol32 RBM peptides in the presence of non-radioactive ATP for 2 h and analysed by mass spectrometry (n = 1). Lollipop plots show phosphorylation events, which occurred exclusively within the thioredoxin tag. PSM, peptide-spectrum match. Source data are provided as a Source Data file.

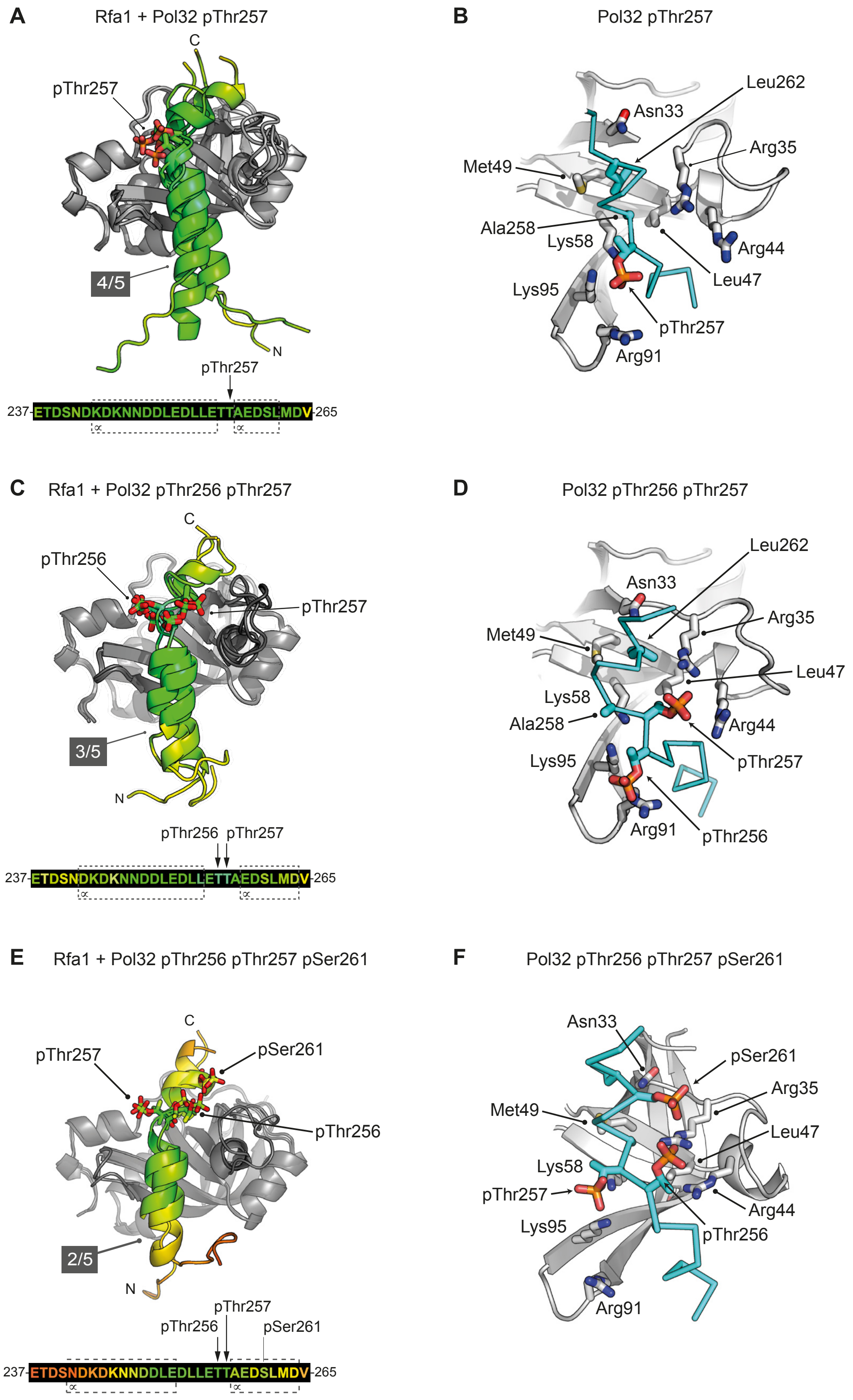

Supplementary Figure 5 | Molecular models for Pol32 phospho-RBM-RPA interactions. (A) Superposition of AlphaFold models for Rfa1 NTD in complex with the Pol32 RBM phosphorylated at Thr257, and (B) molecular cartoon representation of the predicted interface. (C) Superposition of AlphaFold models for Rfa1 NTD in complex with the Pol32 RBM

phosphorylated at Thr256 and Thr257, and (D) molecular cartoon representation of the predicted interface. (E) Superposition of AlphaFold models for Rfa1 NTD in complex with the Pol32 RBM phosphorylated at Thr256, Thr257, and Ser261, and (F) molecular cartoon representation of the predicted interface. Models in panels A, C, and E are shown in cartoon representation with Rfa1 NTD rendered grey and the identified interacting regions of Pol32 coloured according to pLDDT (predicted Local Distance Difference Test) score, using a continuous rainbow spectrum from red (low confidence) to blue (high confidence). The number of models superposed is indicated in each case (grey box), representing those with high similarity and consistency. Insets show the amino acid sequences of the respective Rfa1 NTD-interacting regions with α-helical segments predicted with high consistency indicated with a dotted outline and labelled ‘α’.

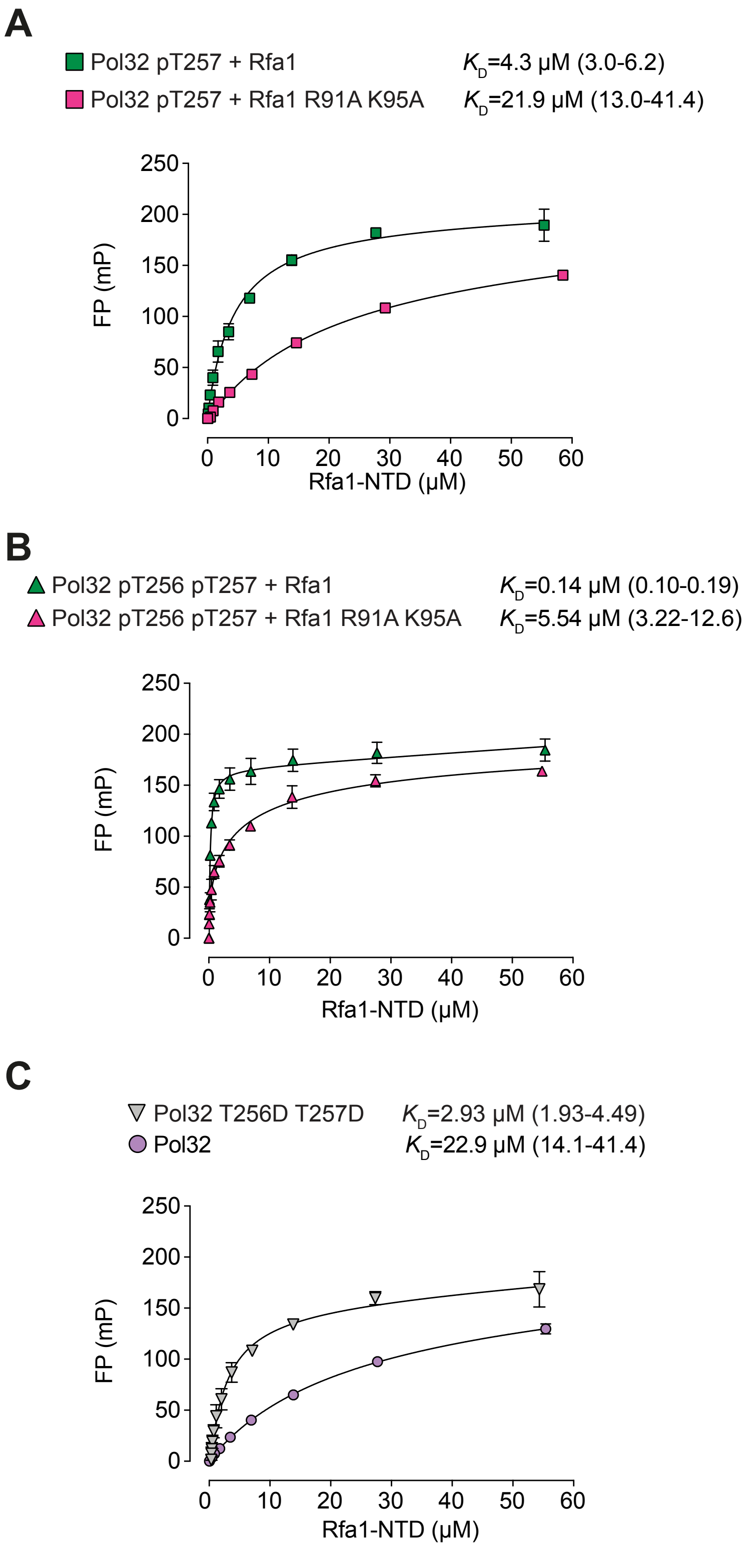

Supplementary Figure 6 | Protein interactions between Pol32 RBM peptides and wild-type or mutant Rfa1 NTD protein. (A, B) Fluorescence polarization (FP) measurements demonstrating binding between the Rfa1 NTD and the indicated fluorescein-labelled Pol32 RBM peptides phosphorylated at Thr257 or Thr256/257, or (C) harbouring phospho-mimetic mutations T256D and T257D, with calculated dissociation constants (K_D) (mean ± SD, with uncertainty in the mean provided by 95% confidence intervals in brackets; n = 3 technical replicates). The wild-type Pol32 RBM peptide is included as reference. Source data are provided as a Source Data file.

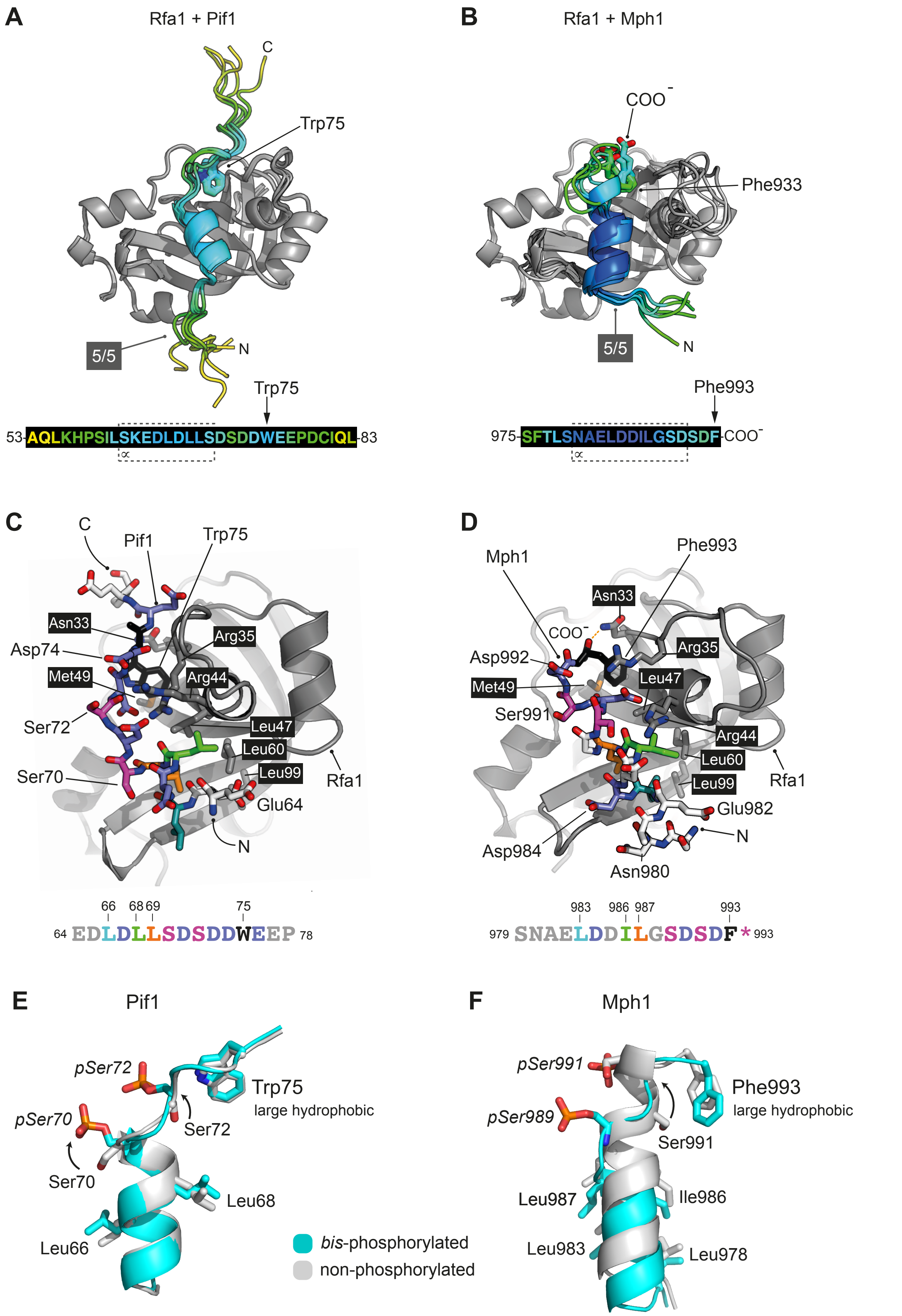

Supplementary Figure 7 | Molecular models for Pif1- and Mph1-RPA interactions. (A) Superposition of AlphaFold models for Rfa1 NTD in complex with the Pif1 RBM and (B) the newly defined Mph1 RBM. Models are shown in cartoon representation with Rfa1 NTD rendered grey and the identified interacting regions of Pif1 and Mph1 coloured according to

pLDDT (predicted Local Distance Difference Test) score, using a continuous rainbow spectrum from red (low confidence) to blue (high confidence). The number of models superposed is indicated in each case (grey box), representing those with high similarity and consistency. Insets show the amino acid sequences of the respective Rfa1 NTD-interacting regions with α-helical segments predicted with high consistency indicated with a dotted outline and labelled 'α'. (C, D) Molecular cartoon representations of the predicted interface between Rfa1 NTD and Pif1 RBM, and Rfa1 NTD and Mph1 RBM, respectively. Key amino acids are shown in stick representation and are colour-coded as per main Fig. 4E. (E) Superposition of representative AlphaFold models for Pif1 RBM in both non-phosphorylated and *bis*-phosphorylated forms. Only small local movements are observed, primarily in the phosphorylated serine residues (as indicated by arrows). Selected amino acids are shown in stick representation. See key for additional information. (F) As panel E, but for Mph1-RBM.

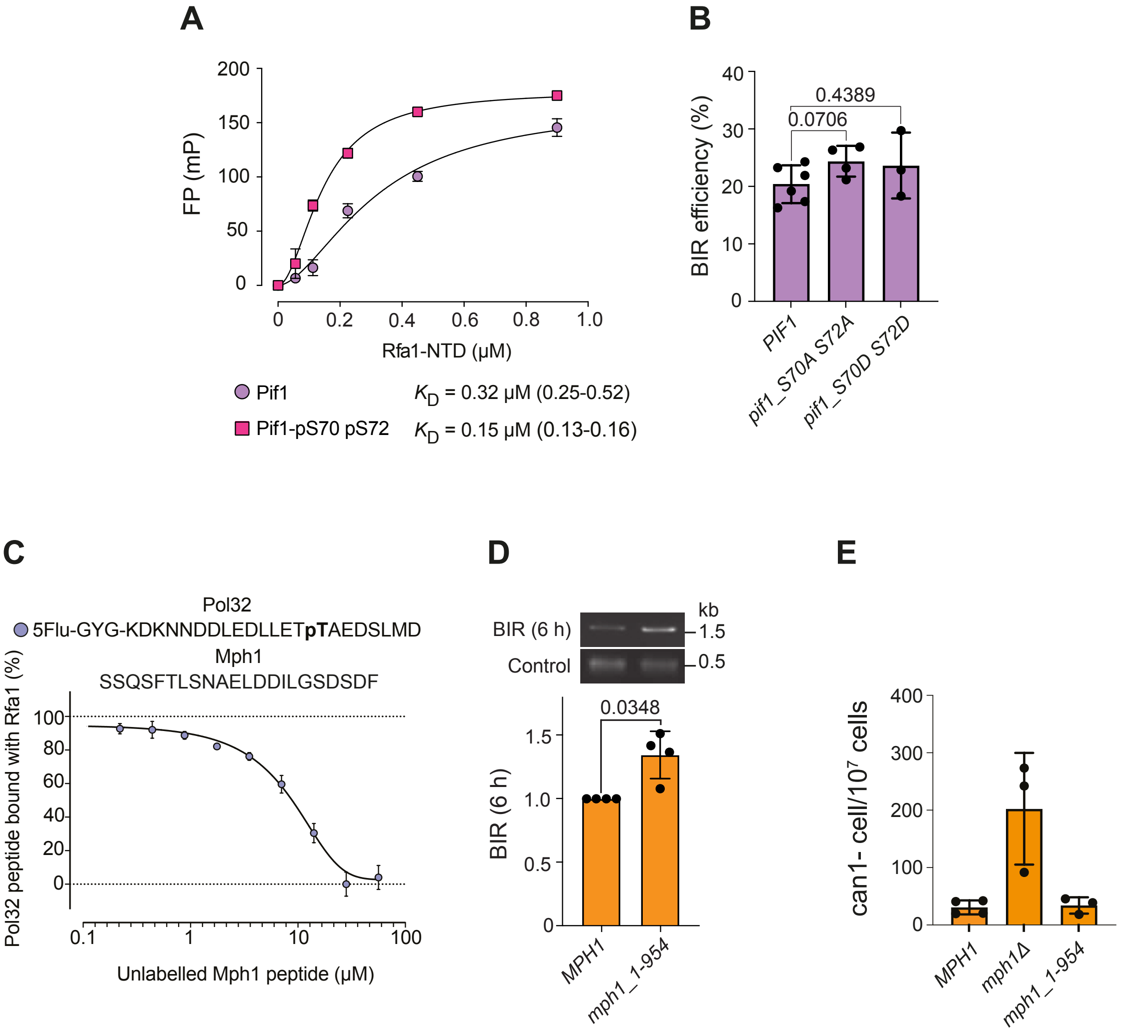

Supplementary Figure 8 | Investigation of the Pif1 and Mph1 RBMs. (A) Fluorescence polarization (FP) experiment demonstrating binding between a fluorescein-labelled Pif1 RBM peptide phosphorylated at Ser70/72 and the Rfa1 NTD, and calculated dissociation constants (K_D) (mean ± SD, with uncertainty in the mean provided by 95% confidence intervals in brackets; n = 3 technical replicates). The wild-type Pif1 RBM peptide is included as reference. (B) BIR efficiency measurements for strains harbouring wild-type Pif1 and variants with phospho-null or phospho-mimetic amino substations at Ser70/72 (mean ± SD; *PIF1*, n = 6; *pif1_S70A S72A*, n = 4; *pif1 _S70D S72D*, n = 3 independent biological repeats). Statistical significance of differences in BIR efficiency between strains was assessed by two-tailed Welch's t-test with *p* values indicated. (C) Competition FP experiment between the indicated Pol32 (fluorescently labelled) and Mph1 RBM peptides (mean ± SD; n = 3 technical replicates). The fitted line is shown for illustrative purposes only. (D) PCR-based BIR measurements for the indicated strains 6 h after HO endonuclease induction by addition of galactose to the culture medium (mean ± SD; n = 4 independent biological repeats). The positions of size markers (kb) are indicated. The BIR to control ratio determined for the strain expressing wild-type *MPH1 was* set to 1. Statistical significance of differences in BIR efficiency was assessed by two-tailed Welch's t-test with *p* values indicated. (E) Loss of Mph1 results in a mutator phenotype, measured here as an increase in spontaneous loss-of-function mutations within the *CAN1* gene (mean ± SD; *MPH1*, n = 4; *mph1Δ*, n = 3; *mph1_1-954, n = 3* independent biological repeats). Statistical significance of differences in mutation frequencies between strains was assessed by two-tailed Welch's t-test with *p* values indicated. Removal of the RBM from Mph1 is not associated with overtly increased mutations, showing that Mph1_1-954 remains functional. Source data are provided as a Source Data file.

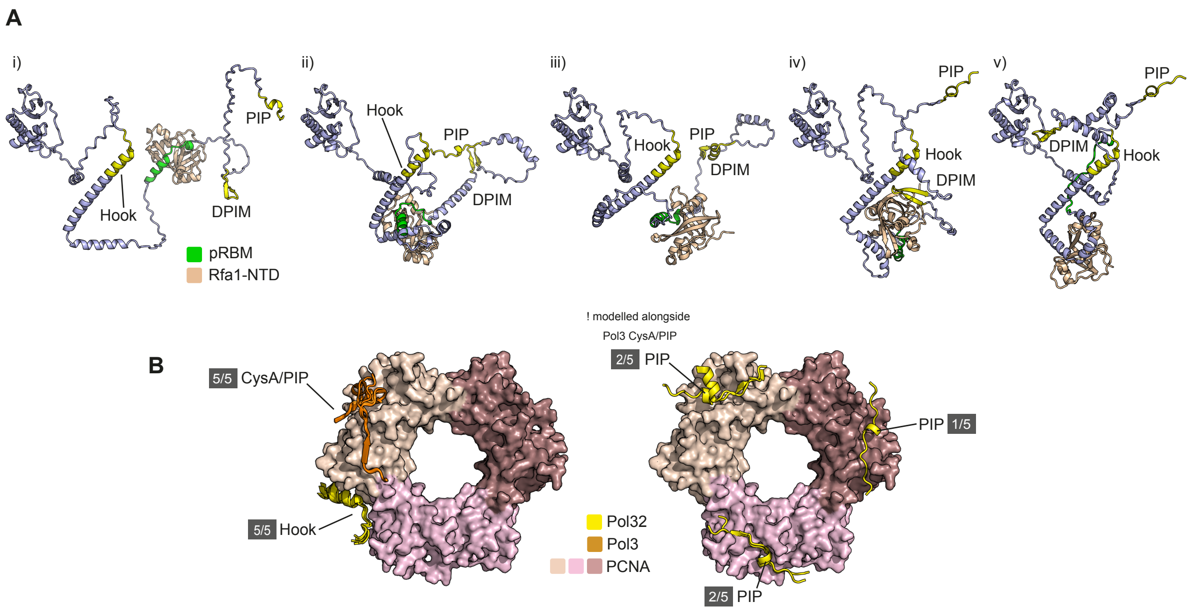

Supplementary Figure 9 | Molecular models for Pol32 interactions with RPA and PCNA in the context of the Pol δ-PCNA-DNA complex. (A) Molecular cartoon representations for full-length *bis*-phosphorylated Pol32 (pRBM), extracted from the five AlphaFold models (labelled i to v) generated for Pol δ in complex with a primer-template junction, PCNA and Rfa1 NTD (see Fig. 5, main text). Component motifs (Hook, DPIM and PIP-box) are labelled and coloured yellow. See key for additional information. (B) *Left*, both the CysA/PIP motif of Pol3 and the 'Hook' motif from Pol32 are consistently positioned with respect to each other, in complex with a hydrophobic pocket/IDCL and a monomer-monomer interface of the PCNA homotrimer, respectively (5 out of 5 models). *Right*, the Pol32 PIP-box is consistently modelled as bound to one of the three available hydrophobic pocket/IDCL interfaces of PCNA. In two models, AlphaFold co-locates the Pol32 PIP-box and Pol3-CysA/PIP bound to the same pocket; however, we suspect this may be a modelling artefact. See key for additional information.

Supplementary Table 1: *S. cerevisiae* strains used in this study (name and genotype).

BY4741	*MATa his3Δ1 leu2Δ0 met15Δ0 ura3Δ0*
YRL620	BY4741 *POL32-9MYC (kanMX)*
YRL621	BY4741 *pol32_1-333-9MYC (kanMX)*
YRL622	BY4741 *pol32_1-269-9MYC (kanMX)*
YRL623	BY4741 *pol32_1-249-9MYC (kanMX)*
YRL624	BY4741 *POL32-3FLAG pep4Δ*
YRL625	BY4741 *POL31-2TAP POL32-3FLAG pep4Δ*
YRL626	BY4741 *mph1Δ (hphNT1)*
YRL627	BY4741 *mph1_1-954-9MYC (HIS3MX)*
JRL092	*MATa::HOcsΔ::hisG ura3Δ851 trp1Δ63 sup53Δ::leu2Δ::kanMX hmlΔ:: hisG HMRa-stk ade3::GAL10::HO can1,1-1446::HOcs::hphNT ykl215c::LEU2::canΔ289*
YRL629	JRL092 *pol32_1-333 (natNT2)*
YRL630	JRL092 *pol32_1-269 (natNT2)*
YRL631	JRL092 *pol32_1-249 (natNT2)*
YRL632	JRL092 *pol32Δ (natNT2)*
YRL637	JRL092 *pol32_* ΔRBM (Δ*N247-A258*)
YRL638	JRL092 *pol32_*RBM* (*L253R A258R)*
YRL639	JRL092 *pol32_1-333 RBM** (*L253R A258R)*
YRL646	JRL092 *pol32_T256D T257D*
YRL647	JRL092 *mph1Δ (natNT2)*
YRL648	JRL092 *mph1_1-954-9MYC (natNT2)*
pJ69-4A	*ade2-1 his3-200 leu2-3,112 trp1-901 ura3-52 gal4Δ gal80Δ MET2::GAL7-lacZ LYS2::GAL1-HIS3 GAL2-ADE2*
YRL649	pJ69-4A *pGBKT7-∅ (TRP1+) pGADT7-∅ (LEU2+)*
YRL650	pJ69-4A *pGBKT7-POL32 (TRP1+) pGADT7-∅ (LEU2+)*
YRL651	pJ69-4A *pGBKT7-∅ (TRP1+) pGADT7-POL30 (LEU2+)*
YRL652	pJ69-4A *pGBKT7-POL32 (TRP1+) pGADT7-POL30 (LEU2+)*
YRL653	pJ69-4A *pGBKT7-pol32_1-333 (TRP1+) pGADT7-POL30 (LEU2+)*
YRL654	pJ69-4A *pGBKT7-∅ (TRP1+) pGADT7-POL1 (LEU2+)*
YRL655	pJ69-4A *pGBKT7-POL32 (TRP1+) pGADT7-POL1 (LEU2+)*
YRL656	pJ69-4A *pGBKT7-pol32_1-269 (TRP1+) pGADT7-POL1 (LEU2+)*
YRL661	BY4741 *POL31-2TAP (hphNT1) pol32 (248-259Δ)-3FLAG (kanMX) pep4Δ (URA3)*
YRL662	JRL092 *pol32-T256A T257A*
YRL663	JRL092 *pol32 (1-333)-T256D T257D*
YRL664	JRL092 *pol32 (1-333)-T256A T257A*
YRL665	JRL092 *PIF1::pif1 (65-72Δ)*
YRL666	JRL092 *MPH1::mph1-F993A*
AM1003	*MATa-LEU2-tel/MATa-inc ade1 met13 ura3 leu2-3,112/leu2 thr4 lys5 hml::ADE1/hml::ADE3 hmr::HYG ade3::GAL-HO FS2::NAT/FS2*
YRL667	AM1003 *pol32Δ (kanMX)*
YRL668	AM1003 *POL32::pol32 (1-333) (kanMX)*
YRL669	AM1003 *POL32::pol32 (1-249) (kanMX)*
YRL670	BY4741 *CLB1::CLB1-TAP (HIS3MX)*
YRL671	BY4741 *CLB3::CLB3-TAP (HIS3MX)*
YRL672	BY4741 *CLB5::CLB5-TAP (HIS3MX)*
YRL673	BY4741 *CDC28::CDC28-TAP (HIS3MX)*
YRL674	BY4741 *CDC5::CDC5-TAP (HIS3MX)*
YRL675	BY4741 *CDC15::CDC15-TAP (HIS3MX)*
YRL676	BY4741 *CKA2::CKA2-TAP (HIS3MX)*
YRL677	BY4741 *RIM11::RIM11-TAP (HIS3MX)*
YRL678	BY4741 *CDC7::CDC7-TAP (HIS3MX)*
YRL679	JRL092 *PIF1::pif1-S70A S72A*
YRL680	JRL092 *PIF1::pif1-S70D S72D*

Supplementary Table 2: Synthetic peptides used in this study. Position of phosphorylated Pol32 amino acid residues is indicated in the sequence using square brackets.

**Peptide [UNIPROT ID]**	**Amino acids**	**Sequence**
Pol32 [DPOD3_YEAST]	243-264	KDKNNDDLEDLLETTAEDSLMD
Pol32 RBM*	243-264	KDKNNDDLEDRLETTREDSLMD
Pol32 pThr257	243-264	KDKNNDDLEDLLET[pT]AEDSLMD
Pol32 pThr256 pThr257	243-264	KDKNNDDLEDLLE[pT][pT]AEDSLMD
Pol32 T256D T257D	243-264	KDKNNDDLEDLLEDDAEDSLMD
Pol32 pThr256 pThr257 pSer261	243-264	KDKNNDDLEDLLE[pT][pT]AED[pS]LMD
Pif1 [PIF1_YEAST]	59-79	SILSKEDLDLLSDSDDWEEPD
Pif1 pSer70 pSer72	59-79	SILSKEDLDLL[pS]D[pS]DDWEEPD
Mph1 [MPH1_YEAST]	972-993	SSQSFTLSNAELDDILGSDSDF
